# Current status of artificial intelligence analysis for the treatment of pancreaticobiliary diseases using endoscopic ultrasonography and endoscopic retrograde cholangiopancreatography

**DOI:** 10.1002/deo2.267

**Published:** 2023-06-30

**Authors:** Takamichi Kuwahara, Kazuo Hara, Nobumasa Mizuno, Shin Haba, Nozomi Okuno, Toshitaka Fukui, Minako Urata, Yoshitaro Yamamoto

**Affiliations:** ^1^ Department of Gastroenterology Aichi Cancer Center Hospital Aichi Japan

**Keywords:** artificial intelligence, deep learning, EUS, pancreas, ERCP

## Abstract

Pancreatic and biliary diseases encompass a range of conditions requiring accurate diagnosis for appropriate treatment strategies. This diagnosis relies heavily on imaging techniques like endoscopic ultrasonography and endoscopic retrograde cholangiopancreatography. Artificial intelligence (AI), including machine learning and deep learning, is becoming integral in medical imaging and diagnostics, such as the detection of colorectal polyps. AI shows great potential in diagnosing pancreatobiliary diseases. Unlike machine learning, which requires feature extraction and selection, deep learning can utilize images directly as input. Accurate evaluation of AI performance is a complex task due to varied terminologies, evaluation methods, and development stages. Essential aspects of AI evaluation involve defining the AI's purpose, choosing appropriate gold standards, deciding on the validation phase, and selecting reliable validation methods. AI, particularly deep learning, is increasingly employed in endoscopic ultrasonography and endoscopic retrograde cholangiopancreatography diagnostics, achieving high accuracy levels in detecting and classifying various pancreatobiliary diseases. The AI often performs better than doctors, even in tasks like differentiating benign from malignant pancreatic tumors, cysts, and subepithelial lesions, identifying gallbladder lesions, assessing endoscopic retrograde cholangiopancreatography difficulty, and evaluating the biliary strictures. The potential for AI in diagnosing pancreatobiliary diseases, especially where other modalities have limitations, is considerable. However, a crucial constraint is the need for extensive, high‐quality annotated data for AI training. Future advances in AI, such as large language models, promise further applications in the medical field.

## INTRODUCTION

Pancreatic and biliary diseases encompass a wide range of conditions. The pancreatic conditions include cystic lesions such as intraductal papillary mucinous neoplasm (IPMN) and mucinous cystic neoplasm; neoplastic lesions such as pancreatic ductal adenocarcinoma and pancreatic neuroendocrine tumor; and inflammatory lesions such as chronic pancreatitis and autoimmune pancreatitis (AIP).^1^, ^2^, ^3^, ^4^ Similarly, biliary diseases include neoplastic lesions, such as cholangiocarcinoma, and inflammatory conditions, such as primary sclerosing cholangitis and immunoglobulin 4‐related sclerosing cholangitis. Accurate diagnosis before treatment is essential, as treatment strategies differ significantly for each disease. Various imaging modalities such as computed tomography, magnetic resonance imaging, abdominal ultrasonography, endoscopic ultrasonography (EUS), and endoscopic retrograde cholangiopancreatography (ERCP) are used to diagnose diseases in the hepatopancreatobiliary region. High‐resolution images of the pancreas and biliary tract can be obtained using EUS, an important modality for treating pancreatobiliary diseases.^5^ Procedures such as contrast‐enhanced EUS (CE‐EUS), EUS‐guided fine needle aspiration/biopsy (EUS‐FNA/B), and EUS‐elastography enhance the diagnostic performance of EUS.^6^, ^7^, ^8^, ^9^, ^10^, ^11^ However, EUS alone cannot diagnose all pancreatobiliary diseases because of its low specificity, even when using EUS‐related procedures (with 80%–95% accuracy).^12^ ERCP is also used for the diagnosis of pancreatic and biliary tract diseases and enables simultaneous interventions such as stone removal and bile duct stenting. However, it might result in severe adverse events such as post‐ERCP pancreatitis.^13^

Artificial intelligence (AI) is a mathematical classification or regression technique, while “deep learning” is an AI algorithm and an advanced machine learning method that uses neural networks.^14^ During the past decade, AI has made dramatic progress and has been applied in the medical field, including for the diagnosis of pancreatobiliary diseases using numerous types of modalities.^11^, ^12^, ^15^, ^16^, ^17^, ^18^, ^19^ However, most of the associated reports have not been systematically categorized. This review describes two columns: 1) a simple checklist for evaluating AI performance and 2) the current status of AI for EUS and ERCP, especially related to pancreatobiliary diseases. However, this article is neither a systematic review nor a meta‐analysis, as the published database was not systemically researched for publication as a meta‐analysis.

## MACHINE LEARNING AND DEEP LEARNING

Several AI architectures exist, including machine learning (ML) and deep learning (DL). Although DL is a subset of ML, there is a clear difference between the two in terms of whether feature extraction (such as texture analysis and histogram analysis) and feature selection (such as filter method and wrapper method) are performed during the preprocessing stage. DL does not require feature extraction and selection because it can directly use images as input values.^14^, ^20^

Various architectures have been employed to develop ML, including support vector machines, decision trees, random forests, factorization machines, logistic regression analyses, and neural networks (NN).^21^, ^22^ Gradient boosting machines are an evolution of random forests. Convolutional NNs or transformer architectures are utilized. Deep learning is generally utilized when images are used as input values. In comparison, radiomics generally refers to the process of performing feature extraction and selection on images, which are then input into ML rather than DL.^23^

The primary roles of AI in the medical field include imaging diagnosis, so‐called computer‐aided diagnosis, and lesion detection, so‐called computer‐aided detection systems. The computer‐aided detection system can be further classified into object detection and image segmentation.

## EVALUATION METHOD OF AI PERFORMANCE

There are numerous articles on medical AI, but their findings are different. In addition, there are many specific terms about AI and some evaluation methods, metrics, and development phases, unlike general clinical medical research. Therefore, these factors may make it difficult to evaluate AI performance appropriately. Although guidelines such as standards for Reporting Diagnostic accuracy studies and transparent reporting of a multivariable prediction model for individual prognosis or diagnosis are important references, it is crucial to consider some checkpoints (design, input value, data volume, model, and metrics) for evaluating AI performance, as these guidelines can be somewhat complex.^24^, ^25^

### Design

**FIGURE 1:**
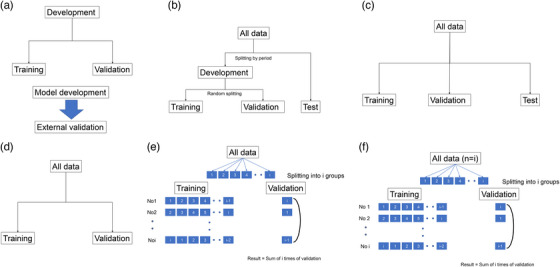
Data splitting method during artificial intelligence (AI) development and validation. (a) External validation: Split the development data into training data and validation data. External validation data was collected from the cohort independent of the development data (e.g., data from other institutions or data collected after AI development). (b) Split‐sample validation. the collected data were randomly divided into training, validation, and test sets. (c) Temporary validation. All data are divided into development and test data by period. Development data is randomly divided into training and validation data. (d) Internal validation (hold‐out method). All data is randomly divided into training and validation data. (e) Internal validation (k‐fold cross‐validation). k‐fold cross‐validation divides the dataset into k groups. One group is used for validation, while the others are used for training the model. This process is repeated k times, and the results are averaged. (f) Internal validation (leave‐one‐out validation). A model is created by dividing one case of a cohort into a validation group and the others into a training group, and after training validation, the same procedure is repeated so that all data are in the validation group, and the validation result of all cases is the final result.

First, we need to confirm the type of AI developed for classification, detection, or other purposes. The gold standard for the labels of each image should be checked. A pathological diagnosis or commonly used diagnostic criteria are desirable as the gold standard; however, in benign diseases where the pathological diagnosis is difficult, a combination of pathological findings and clinical observations (no malignant findings on biopsy and no change with follow‐up) is acceptable. It is important to confirm whether the labels are for binary or multiclass classification and to check the definition of each label. For binary classification, it is important to confirm the definition of the control group. Next, we need to confirm the validation phase (internal or external). The recommended approach for external validation is to randomly divide the collected data into training and validation sets and collect a test set from another facility after developing the model (Figure 1a). If it is difficult to collect a separate test set, another option is to perform split‐sample validation by randomly dividing the collected data into three sets: training, validation, and test (Figure 1b). However, this approach does not strictly qualify as external validation but rather as internal validation. Therefore, employing a method called temporal validation is preferable, in which the test set is distinguished from the training and validation sets by setting a specific time period (Figure 1c).^24^, ^25^ Subsequently, the validation methods (cross‐validation or holdout) should be confirmed during the internal validation phase (Figure 1d). Several types of cross‐validation methods exist, including leave‐one‐out and K‐fold cross‐validation. K‐fold cross‐validation is a method in which the entire dataset is divided into smaller chunks or folds called K groups. One group is used as the validation set, and the remaining groups are used as the training set to build the model. This process is repeated K times, with each group serving as the validation set. The results are obtained by averaging the validation results from all the iterations (Figure 1e). In leave‐one‐out, each data point in the dataset is considered as the validation set once, whereas the remaining data points are used for training. This means that if there are ‘n’ data points, the model is trained and validated ‘n’ times^24^, ^25^ (Figure 1f).

### Input value and models

The type of input values (image, clinical features, or a combination of these) should be confirmed. Next, it is necessary to determine whether the method is based on DL or ML. In the case of ML, it is important to consider the feature extraction methods, such as histograms or texture analysis.^24^, ^25^

### Data volume

The inclusion criteria for the target cases and the method used for data splitting should be verified. DL often requires more training data than ML (DL > 1000, ML > 100).^5^, ^20^ To prevent data leakage (a phenomenon where information from the training group seeps into the validation/test groups, making it seem more accurate), all data from the same cases should be in the same split groups when the data are divided.

### Metrics

Subsequently, the evaluation metrics of the model should be assessed. For classification tasks (such as disease diagnosis), the accuracy, sensitivity, specificity, and area under the curve from receiver operating characteristic (ROC) analysis can be used. In this case, describing the results using a confusion matrix is desirable. Detection performance can be evaluated for detection tasks using metrics such as intersection over union (IoU) or Dice score. Generally, successful detection is defined as IoU≥0.5.^26^ The IoU and confidence scores are used to classify true positives (correctly detected), false positives (misdetected), and false negatives (detection failed). Unlike in classification problems, true negatives (non‐lesion part not detected) cannot be theoretically calculated because there are countless negative areas. Therefore, detection evaluation metrics often include sensitivity (recall) or positive predictive value (precision), area under the curve of the precision‐recall‐curve, which is the average precision, and the mean average precision across classes. Theoretically, specificity, negative predictive value, and accuracy are not used (although calculations are sometimes performed by considering images without lesions as negative samples).^5^, ^20^ Metrics such as the mean IoU across classes are used for the segmentation tasks. Essentially, the metrics of the external validation group are the primary endpoints of the report. In the case of internal validation, the results of the validation group in the holdout method and the results of all cases in the cross‐validation become the primary endpoints.

## AI FOR EUS IMAGES

While referring to existing articles on AI in the field of biliary and pancreatic medicine, the PubMed, Embase, and Cochrane databases were systematically searched for articles published from inception to March 31, 2023, by one author (Takamichi Kuwahara).^5^, ^20^, ^27^, ^28^, ^29^ The search terms used were as follows: (artificial intelligence OR deep learning OR machine learning OR radiomics) AND (endoscopic ultrasonography OR endoscopic ultrasound OR EUS OR ERCP OR cholangioscopy). The findings of these articles indicated that AI for EUS has been developed for numerous purposes, such as classifying and detecting pancreatic tumors, pancreatic cysts, and submucosal tumors.

### Detection of pancreatic tumors

One article on the AI detection of pancreatic tumors from EUS images was reported by Tonozuka et al. (Table 1).^30^ They developed an AI to detect pancreatic tumors from EUS images using a fully convolutional network based on a convolutional NN with EUS images from 93 cases of pancreatic disease. When their accuracy was evaluated using 47 test data, they reported an area under the ROC curve of 0.94.

**TABLE 1:** Main characteristics of included studies about endoscopic ultrasonography‐artificial intelligence (EUS‐AI) for pancreatic tumors.

Authors	Design		Input	Model	Data	Metrics
Pancreatic tumors						
Tonozuka^30^	Detection (PDAC)	Internal 3‐split	EUS images	DL	93/47	AUROC 0.94
Zang^31^	Classification (PDAC/normal)	Internal LOO	EUS images	ML SVM	216	Accuracy 0.979
Das^32^	Classification (PDAC/normal)	Internal	EUS features	ML SVM	56	AUROC 0.93
Norton^33^	Classification (PDAC/CP)	Only train data	EUS features	ML SVM	21	Accuracy 0.89
Zhu^34^	Classification (PDAC/CP)	Internal hold‐out	EUS features	ML SVM	398	Accuracy 0.942
Saftiou^35^	Classification (PDAC/CP)	Internal 3‐fold CV	CE‐EUS	ML NN	167	Sensitivity 0.944
Ozkan^36^	Classification (PDAC/normal)	Internal hold‐out	EUS features	ML NN	332/100	Accuracy 0.875
Saftiou^37^	Classification (PDAC/CP)	Internal 10‐fold CV	EUS‐EG	ML NN	69	Accuracy 0.897
Saftiou^38^	Classification (PDAC/CP)	Internal 10‐fold CV	EUS‐EG	ML NN	258	Accuracy 0.843
Kuwahara^12^	Classification (Carcinoma/non‐carcinoma)	Temporary	EUS images	DL	772/161	Accuracy 0.91
Maraya^39^	Classification (PDAC/AIP)	Internal 3‐split	EUS images	DL	460/123	Sensitivity 0.90
Naito^40^	Classification (PDAC/normal)	External	EUS‐FNB specimen	DL	372/120	Accuracy 0.941
Ishikawa^41^	EUS‐FNB specimen quantity	Internal 8‐fold CV	EUS‐FNB specimen	DL	159	Accuracy 0.844

Abbreviations: AI, artificial intelligence; AIP, autoimmune pancreatitis; AUROC, area under the receiver operating characteristic curve; CE‐EUS, contrast enhanced‐EUS; CP, chronic pancreatitis; CV, cross‐validation; DL, deep learning; EUS, endoscopic ultrasonography; EUS‐EG, EUS‐elastography; EUS‐FNB, EUS–guided fine needle biopsy; LOO, leave‐one‐out; ML, machine learning; NN, neural networks; PDAC, pancreatic ductal carcinoma; SVM, support vector machines.

### Classification of pancreatic tumors

**FIGURE 2:**
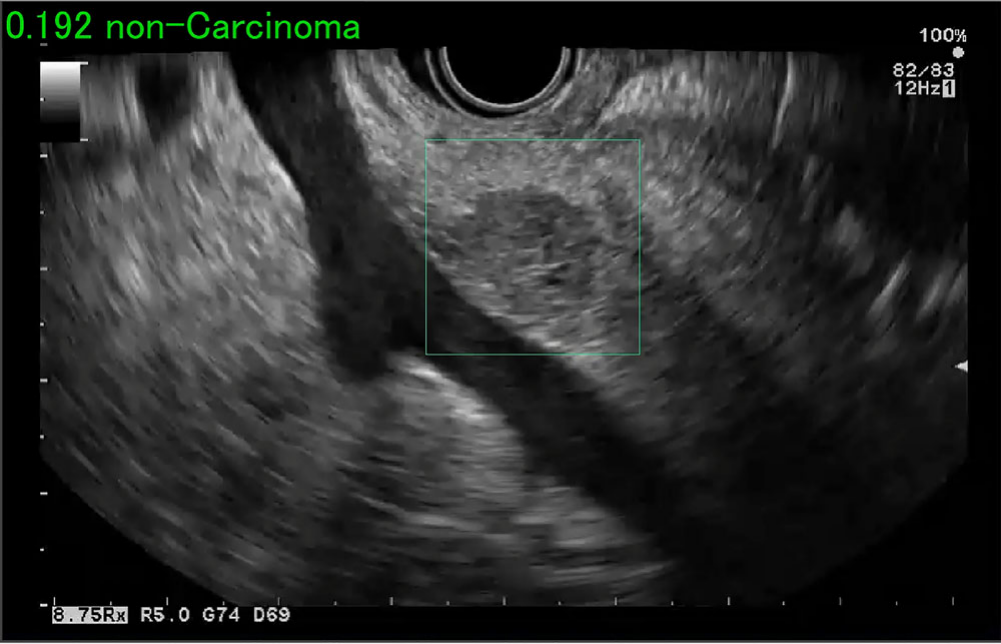
Artificial intelligence image for differential diagnosis of pancreatic masses. Endoscopic ultrasonography image (pseudo papillary neoplasms) is used for the diagnosis of carcinoma by artificial intelligence. The probability of one endoscopic ultrasonography image is expressed in the upper left, and AI diagnoses this lesion as non‐carcinoma.

Twelve articles have been published on AI for classifying pancreatic tumors from EUS images. (Table 1)^12^, ^31^, ^32^, ^33^, ^34^, ^35^, ^36^, ^37^, ^38^, ^39^, ^40^, ^41^ Saftiou et al. extracted features by performing a histogram analysis using EUS‐elastography images of 258 pancreatic cancer or chronic pancreatitis cases and created an AI using NN. They conducted 10‐fold cross‐validation to evaluate its accuracy and reported an accuracy of 0.843.^37^, ^38^ Kuwahara et al. created an AI for diagnosis using EfficientNetV2‐L, one of the classification architectures of DL, with EUS images from 772 cases of multiple pancreatic diseases such as pancreatic cancer, AIP, neuroendocrine tumors (NET), solid‐pseudopapillary neoplasm, and chronic pancreatitis. They evaluated its accuracy using 161 test data and reported an accuracy of 0.91 (Figure 2).^12^ Marya et al. created an AI using ResNet50v2, one of the classification architectures of DL, with EUS images from 460 cases of pancreatic diseases, such as pancreatic cancer, AIP, and chronic pancreatitis. They evaluated its accuracy using 123 test data and reported a sensitivity of 0.9 and specificity of 0.85 (AIP vs. others).^39^

Naito et al. conducted a study in which they annotated pathological images obtained from EUS‐FNA specimens of 372 cases of pancreatic cancer. They used EfficientNet, another classification architecture of DL, for a differential diagnosis of pancreatic cancer. They evaluated the accuracy of the model using 120 external validation data and reported an accuracy of 0.941.^40^ Ishikawa et al. created an AI model to evaluate the quality of EUS‐FNA specimens using macroscopic images of 159 cases of pancreatic cancer. They used ResNet34, one of the classification architectures of DL, and evaluated the accuracy of the model using 8‐fold cross‐validation, reporting an accuracy of 0.844 for pathological diagnosis.^41^

### Classification of pancreatic cysts

**FIGURE 3:**
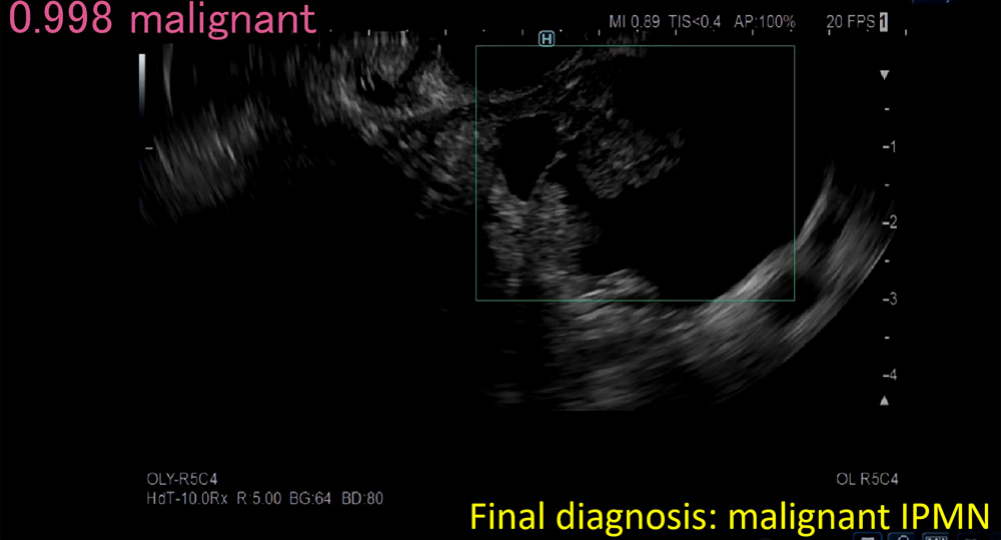
Artificial intelligence image for differential diagnosis of pancreatic cyst. Endoscopic ultrasonography image (intraductal papillary mucinous neoplasms, IPMN) is used for the diagnosis of malignancy by artificial intelligence. The probability of one endoscopic ultrasonography image is expressed in the upper left, and artificial intelligence diagnoses this lesion as malignant.

Five articles on AI detection of pancreatic tumors from EUS images have been reported (Table 2).^42^, ^43^, ^44^, ^45^ Kuwahara et al. created an AI to differentiate the benign‐malignant diagnosis of IPMN using ResNet50, another classification architecture of DL, with EUS images from 50 cases of IPMN.^42^ They conducted 10‐fold cross‐validation to evaluate its accuracy and reported an accuracy of 0.94. The diagnostic ability of the AI was higher than the preoperative diagnostic ability of the doctors (accuracy, 0.58) and the EUS image findings (accuracy, 0.52–0.68; Figure 3). Kurita et al. created an AI to differentiate the benign‐malignant diagnosis of pancreatic cysts using an original DL algorithm with two hidden layers, analysis of cyst fluid, cyst fluid cytology, clinical information, and blood test results from 85 cases of pancreatic cysts.^43^ They conducted 5‐fold cross‐validation to evaluate its accuracy and reported an accuracy of 0.93. The accuracy of the AI was significantly higher than that of carcinoembryonic antigen in cystic fluid and cytology. Machicado et al. created an AI to differentiate the benign‐malignant diagnosis of IPMN using VGG16 and a faster‐R‐convolutional NN, one of the classification and segmentation architectures of DL, with EUS‐guided needle‐based confocal laser endomicroscopy images from 35 cases of IPMN. They conducted 5‐fold cross‐validation to evaluate its accuracy and reported an accuracy of 0.85. The diagnostic ability of the AI was higher than the high‐risk features according to guidelines (accuracy, 0.68–0.74).^44^

**TABLE 2:** Main characteristics of included studies about endoscopic ultrasonography‐artificial intelligence for other diseases.

Authors	Design		Input	Model	Data	Metrics
Pancreatic cysts						
Kuwahara^42^	Classification (IPMN benign/malignant)	Internal 10‐fold CV	EUS images	DL	50	Accuracy 0.94
Kurita^43^	Classification (pancreatic cysts benign/malignant)	Internal 5‐fold CV	EUS‐FNA (cyst fluid) Clinical features	DL	85	Accuracy 0.93
Machicado^44^	Classification (IPMN benign/malignant)	Internal 5‐fold CV	CLE images	DL	35	Accuracy 0.85
Nguon^45^	Classification (MCN/SCN)	Internal 3‐fold CV	EUS images	DL	109	Accuracy 0.83
Pancreatic parenchyma						
Zang^26^	Detection (pancreatic parenchyma)	External	EUS images	DL	294/20	Sensitivity 0.98
Gallbladder lesions						
Jang^46^	Classification (neoplastic/non‐neoplastic)	External	EUS images	DL	1039/83	Accuracy 0.76
Subepithelial lesions						
Kim^47^	Classification (GIST/non‐GIST)	Internal holdout	EUS images	DL	179/69	Accuracy 0.79
Minoda^48^	Classification (GIST/non‐GIST)	External	EUS images	DL	173/60	Accuracy 0.86
Oh^49^	Classification (GIST/leiomyoma)	Internal holdout	EUS images	DL	168/54	Accuracy 0.92
Seven^50^	Classification (GIST/leiomyoma)	External	EUS images	DL	55/15	Accuracy 0.66
Hirai^51^	Classification (GIST/leiomyoma/schwannoma/NET/ectopic pancreas)	Internal 3‐split	EUS images	DL	509/122	Accuracy 0.86
Tanaka^52^	Classification (GIST/leiomyoma)	Internal LOO	CE‐EUS	DL	53	Accuracy 0.91
Yang^53^	Classification (GIST/leiomyoma)	External	EUS images	DL	702/24	Accuracy 0.66

Abbreviations: AI, artificial intelligence; CLE, confocal laser endoscopy; CV, cross‐validation; DL, deep learning; EUS, endoscopic ultrasonography; EUS‐EG, EUS‐elastography; EUS‐FNA, EUS–guided fine needle aspiration; GIST, gastrointestinal stromal tumors; IPMN, intraductal papillary mucinous neoplasms; LOO, leave‐one‐out; MCN, mucinous cystic neoplasms; ML, machine learning; NET, neuroendocrine tumors; NN, neural networks; PDAC, pancreatic ductal carcinoma; SCN: serious cyst neoplasms; SVM, support vector machines.

### Detection of pancreas parenchyma

Zang et al. created an AI to detect pancreatic parenchyma using UNet++, a segmentation architecture of DL, with 294 EUS images as input values. When they evaluated its accuracy using 20 external validation data, they reported a sensitivity (recall) of 0.984 and a positive predictive value (precision) of 0.824 (Table 2). In the same report, they also created an AI to differentiate the current observation position by dividing the typical observation positions of EUS into six and reported an accuracy of 0.862.^31^

### Classification of gallbladder lesions

Jang et al. created an AI for differentiating neoplastic from non‐neoplastic gallbladder polyps using ResNet50, one of the classification architectures of DL, with EUS images as input values for 1039 cases (Table 2). The accuracy of this model was evaluated using 83 sets of external validation data and was found to be 0.762.^46^

### Classification of subepithelial lesions

**FIGURE 4:**
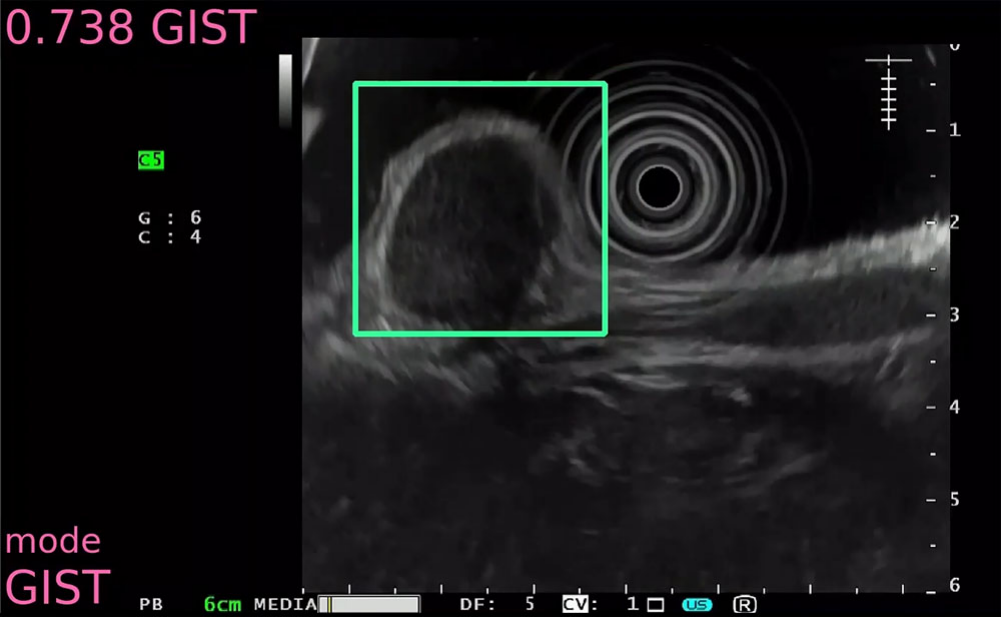
Artificial intelligence image for differential diagnosis of subepithelial lesion. Endoscopic ultrasonography (EUS) image (gastrointestinal stromal tumors, GIST) is used for the differential diagnosis of subepithelial lesions by artificial intelligence. The probability of one EUS image is expressed in the upper left and artificial intelligence diagnoses this lesion as GIST.

Seven articles on AI classification of subepithelial lesions (SELs) from EUS images have been published (Table 2).^47^, ^48^, ^49^, ^50^, ^51^, ^52^, ^53^ Minoda et al. created an AI to differentiate gastrointestinal stromal tumors from leiomyomas using Xception, another classification architecture of DL, with EUS images of 173 cases of SELs. They evaluated its accuracy using 60 test data and reported an accuracy of 0.86 (for lesions <20 mm) and 0.9 (for lesions ≥20 mm). The diagnostic ability of the AI was higher than that of experts and human doctors (accuracy, 0.53–0.73).^48^ Hirai et al. created an AI to diagnose multiclass SELs (gastrointestinal stromal tumors, leiomyoma, schwannoma, NET, and ectopic pancreas) using EfficientNetV2‐L, one of the classification architectures of DL, with EUS images from 509 cases of SELs. They evaluated its accuracy using 122 test data and reported an accuracy of 0.86 (Figure 4). The diagnostic ability of the AI was higher than that of both expert and non‐expert human doctors (accuracy, 0.58).^49^

## AI FOR ERCP

The AI for ERCP was developed for numerous purposes, such as evaluating the difficulty of ERCP using endoscopic images of the duodenal papilla and classifying the benign‐malignant diagnosis of biliary structures using clinical features or cholangioscopy images (Table 3).

**TABLE 3:** Main characteristics of included studies about artificial intelligence (AI) for endoscopic retrograde cholangiopancreatography‐related procedures.

Authors	Design		Input	Model	Data	Metrics
ERCP difficulty evaluation						
Huang^54^	Detection (CBD, stone)	External	Cholangiogram	DL	1381/228	Sensitivity 0.67
Kim^55^	Detection (ampulla)	Internal 5‐fold CV	Endoscopic images	DL	451	mIoU 0.64
Biliary strictures						
Sugimoto^56^	Classification (benign/malignant)	Internal 5‐fold CV	Clinical features	ML GBM	206	Accuracy 0.86
Marya^57^	Classification (benign/malignant)	External	CLE images	DL	122/32	Accuracy 0.90

Abbreviations: AI, artificial intelligence; CBD, common bile duct; CLE, confocal laser endoscopy; CV, cross‐validation; DL, deep learning; ERCP, endoscopic retrograde cholangiopancreatography; GBM, gradient boosting machines; mIoU, mean intersection over units; ML, machine learning.

### Evaluation of the difficulty of ERCP

Two articles on AI evaluation of the difficulty of ERCP have been published (Table 3).^54^, ^55^ Huang et al. developed an AI system using CasNet, a segmentation architecture of DL trained on 1381 cholangiogram images. This AI could detect common bile duct stones. The researchers assessed its accuracy using 228 test data and reported a sensitivity (precision) of 0.67 and a positive predictive value of 0.8. By leveraging this AI, they established a difficulty‐scoring system for stone removal (categorized as difficult or not difficult). They validated the performance of their system using 173 data. The machine lithotripsy rate, treatment time, and stone removal failure rate were significantly higher in cases the AI identified as “difficult” than in those predicted to be “not difficult.”^54^

### Classification of benign‐malignant diagnosis of biliary structures

Sugimoto et al. created an AI to diagnose the malignancy of biliary strictures using lightGBM, a classification architecture of ML, based on clinical and imaging features, such as bile duct diameter, from 206 cases with biliary strictures. They conducted a 5‐fold cross‐validation to evaluate its accuracy and reported an accuracy of 0.86. (Table 3).^56^ Marya et al. created an AI to differentiate the benign‐malignant diagnosis of biliary strictures using ResNet50v2, one of the classification architectures of DL, using cholangioscopy images from 122 cases of biliary strictures. The researchers assessed its accuracy using 32 test data and reported an accuracy of 0.9^57^ (Table 3).

## LIMITATIONS AND FUTURE PERSPECTIVE ON AI FOR EUS AND ERCP

In this review, we evaluated AI for EUS and ERCP and found that it has been developed for numerous purposes. A number of useful reports have been sporadically observed; however, few studies have performed an accurate external validation, resulting in a limited number of reports with high levels of evidence. There are currently no approved biliary or pancreatic AI systems in Japan. The AIs for EUS and ERCP are not as advanced as that for plain endoscopy. One of the major reasons for the development of AI algorithms for EUS and ERCP is the availability of high‐quality annotated data. Training AI models require large datasets encompassing diverse cases; however, EUS and ERCP datasets may be more limited than endoscopy datasets. To overcome this limitation, developing a nationwide system that collects and utilizes EUS and ERCP images is necessary.

AI for EUS and ERCP has been developed only for diagnostic imaging and detection. In recent years, large‐scale language models such as ChatGPT have emerged, and the latest models can use images and audio as input as well as output. Therefore, their regular application in the medical field is anticipated in the future.

## CONCLUSION

The current status, limitations, and future perspectives of AI for EUS and ERCP were reported. The AI has the potential to be a breakthrough in the diagnosis of pancreatobiliary diseases where other modalities have diagnostic limitations.

## CONFLICT OF INTEREST STATEMENT

Takamichi Kuwahara, Kazuo Hara, Shin Haba, Nozomi Okuno, Toshitaka Fukui, Minako Urata, and Yoshitaro Yamamoto declare no conflict of interest related to this study. Nobumasa Mizuno has received Grants or contracts from any entity from to their institution from Novartis, MSD, Incyte, Ono Pharmaceutical, Seagen, Dainippon Sumitomo Pharma; has received payment or honoraria for lectures, presentations, speakers bureaus, manuscript writing or educational events from Yakult Honsha, AstraZeneca, Novartis, FUJIFILM Toyama Chemical, MSD, Taiho Pharmaceutical; and has participated on a Data Safety Monitoring Board or Advisory Board for AstraZeneca.

## ETHICS STATEMENT

This article does not contain any study with human or animal subjects performed by any of the authors.

## References

[1] WHO Classification of Tumours Editorial Board . Digestive System Tumors. WHO Classification of Tumors, 5th edn, Lyon: IARC Press, 2019.

[2] Lloyd RV , Osamura R , Kloppel G , Rosai J . WHO Classification of Tumours of Endocrine Organs, 4th edn, Lyon: IARC Press, 2017.

[3] Okazaki K , Chari ST , Frulloni L *et al*. International consensus for the treatment of autoimmune pancreatitis. Pancreatology 2017; 17: 1–6. 28027896 10.1016/j.pan.2016.12.003

[4] Tanaka M , Fernández‐del Castillo C , Kamisawa T *et al*. Revisions of international consensus Fukuoka guidelines for the management of IPMN of the pancreas. Pancreatology 2017; 17: 738–53. 28735806 10.1016/j.pan.2017.07.007

[5] Kuwahara T , Hara K , Mizuno N *et al*. Current status of artificial intelligence analysis for endoscopic ultrasonography. Dig Endosc 2021; 33: 298–305. 33098123 10.1111/den.13880

[6] Fusaroli P , Napoleon B , Gincul R *et al.* The clinical impact of ultrasound contrast agents in EUS: A systematic review according to the levels of evidence. Gastrointest Endosc 2016; 84: 587–96.e10. 27311654 10.1016/j.gie.2016.06.006

[7] Kuwahara T , Hara K , Mizuno N *et al*. Present status of ultrasound elastography for the diagnosis of pancreatic tumors: Review of the literature. J Med Ultrason (2001) 2020; 47: 413–20. 32444973 10.1007/s10396-020-01026-6

[8] Hirooka Y , Kuwahara T , Irisawa A *et al*. JSUM ultrasound elastography practice guidelines: Pancreas. J Med Ultrasonics 2015; 42: 151–74. 10.1007/s10396-014-0571-7 26576568

[9] Wiresema MJ , Vilmann P , Giovannini M *et al*. Endosonography‐guided fine‐needle aspiration biopsy: Diagnostic accuracy and complication assessment. Gastroenterology 1997; 112: 1087–95. 9097990 10.1016/s0016-5085(97)70164-1

[10] Haba S , Yamao K , Bhatia V *et al*. Diagnostic ability and factors affecting accuracy of endoscopic ultrasound‐guided fine needle aspiration for pancreatic solid lesions: Japanese large single center experience. J Gastroenterol 2013; 48: 973–81. 23090002 10.1007/s00535-012-0695-8

[11] Kurita Y , Kuwahara T , Hara K *et al*. Features of chronic pancreatitis by endoscopic ultrasound influence the diagnostic accuracy of endoscopic ultrasound‐guided fine‐needle aspiration of small pancreatic lesions. Dig Endosc 2020; 32: 399–408. 31361926 10.1111/den.13497

[12] Kuwahara T , Hara K , Mizuno N *et al*. Artificial intelligence using deep learning analysis of endoscopic ultrasonography images for the differential diagnosis of pancreatic masses. Endoscopy 2023; 55: 140–9. 35688454 10.1055/a-1873-7920

[13] Cotton PB , Lehmen G , Vennes J *et al.* Endoscopic sphincterotomy complications and their management: An attempt at consensus. Gastrointest Endosc 1991; 3: 383–93. 10.1016/s0016-5107(91)70740-2 2070995

[14] LeCun Y , Bengio Y , Hinton G . Deep learning. Nature 2015; 521: 436–44. 26017442 10.1038/nature14539

[15] Ting DSW , Cheung CY , Lim G *et al*. Development and validation of a deep learning system for diabetic retinopathy and related eye diseases using retinal images from multiethnic populations with diabetes. JAMA 2017; 318: 2211–23. 29234807 10.1001/jama.2017.18152 PMC5820739

[16] Gulshan V , Peng L , Coram M *et al*. Development and validation of a deep learning algorithm for detection of diabetic retinopathy in retinal fundus photographs. JAMA 2016; 316: 2402–10 27898976 10.1001/jama.2016.17216

[17] Byrne MF , Chapados N , Soudan F *et al*. Real‐time differentiation of adenomatous and hyperplastic diminutive colorectal polyps during analysis of unaltered videos of standard colonoscopy using a deep learning model. Gut 2019; 68: 94–100. 29066576 10.1136/gutjnl-2017-314547 PMC6839831

[18] Hirasawa T , Aoyama K , Tanimoto T *et al*. Application of artificial intelligence using a convolutional neural network for detecting gastric cancer in endoscopic images. Gastric Cancer 2018; 21: 653–60. 29335825 10.1007/s10120-018-0793-2

[19] Horie Y , Yoshio T , Aoyama K *et al*. Diagnostic outcomes of esophageal cancer by artificial intelligence using convolutional neural networks. Gastrointest Endosc 2019; 89: 25–32. 30120958 10.1016/j.gie.2018.07.037

[20] Kuwahara T , Hara K . Literature review of artificial intelligence for the treatment of pancreatobiliary diseases. Nihon Shokakibyo Gakkai Zasshi 2022; 119: 610–25 (Japanese). 35811119 10.11405/nisshoshi.119.610

[21] Cortes C , Vapnik V . Support‐vector networks. Mach Learn 1995; 20: 273–97.

[22] Breiman L . Random forests. Mach Learn 2001; 45: 5–32.

[23] Lambin P , Rios‐Velazquez E , Leijenaar R *et al*. Radiomics: Extracting more information from medical images using advanced feature analysis. Eur J Cancer 2012; 48: 441–6. 22257792 10.1016/j.ejca.2011.11.036 PMC4533986

[24] Collins GS , Reitsma JB , Altman DG *et al*. Transparent Reporting of a multivariable prediction model for Individual Prognosis or Diagnosis (TRIPOD): The TRIPOD statement. Ann Intern Med 2015; 162: 55–63. 25560714 10.7326/M14-0697

[25] Bossuyt PM , Reitsma JB , Bruns DE *et al*. STARD Group. STARD 2015: An updated list of essential items for reporting diagnostic accuracy studies. BMJ 2015; 351: h5527. 26511519 10.1136/bmj.h5527 PMC4623764

[26] Zhang J , Zhu L , Yao L *et al*. Deep learning‐based pancreas segmentation and station recognition system in EUS: Development and validation of a useful training tool (with video). Gastrointest Endosc 2020; 92: 874–85.e3. 32387499 10.1016/j.gie.2020.04.071

[27] Gorris M , Hoogenboom SA , Wallace MB *et al*. Artificial intelligence for the management of pancreatic diseases. Dig Endosc 2021; 33: 231–41. 33065754 10.1111/den.13875 PMC7898901

[28] Chen PT , Chang D , Wu T *et al*. Applications of artificial intelligence in pancreatic and biliary diseases. J Gastroenterol Hepatol 2021; 36: 286–94. 33624891 10.1111/jgh.15380

[29] Zhang B , Zhu F , Li P *et al*. Artificial intelligence‐assisted endoscopic ultrasound in the diagnosis of gastrointestinal stromal tumors: A meta‐analysis. Surgical Endoscopy 2023; 37: 1649–57. 36100781 10.1007/s00464-022-09597-w

[30] Tonozuka R , Itoi T , Nagata N *et al*. Deep learning analysis for the detection of pancreatic cancer on endosonographic images: A pilot study. J Hepatobiliary Pancreat Sci 2020; 28: 95–104. 32910528 10.1002/jhbp.825

[31] Zhang MM , Yang H , Jin ZD *et al*. Differential diagnosis of pancreatic cancer from normal tissue with digital imaging processing and pattern recognition based on a support vector machine of EUS images. Gastrointest Endosc 2010; 72: 978–85. 20855062 10.1016/j.gie.2010.06.042

[32] Das A , Nguyen CC , Li F *et al*. Digital image analysis of EUS images accurately differentiates pancreatic cancer from chronic pancreatitis and normal tissue. Gastrointest Endosc 2008; 67: 861–7. 18179797 10.1016/j.gie.2007.08.036

[33] Norton ID , Zheng Y , Wiersema MS *et al*. Neural network analysis of EUS images to differentiate between pancreatic malignancy and pancreatitis. Gastrointest Endosc 2001; 54: 625–9. 11677484 10.1067/mge.2001.118644

[34] Zhu M , Xu C , Yu J *et al.* Differentiation of pancreatic cancer and chronic pancreatitis using computer‐aided diagnosis of endoscopic ultrasound (EUS) images: A diagnostic test. PLoS One 2013; 8: e63820. 23704940 10.1371/journal.pone.0063820 PMC3660382

[35] Saftoiu A , Vilmann P , Dietrich CF *et al.* Quantitative contrast enhanced harmonic EUS in differential diagnosis of focal pancreatic masses (with videos). Gastrointest Endosc 2015; 82: 59–69. 25792386 10.1016/j.gie.2014.11.040

[36] Ozkan M , Cakiroglu M , Kocaman O *et al.* Age‐based computer‐aided diagnosis approach for pancreatic cancer on endoscopic ultrasound images. Endosc Ultrasound 2015; 5: 101–7. 10.4103/2303-9027.180473 PMC4850788 27080608

[37] Saftoiu A , Vilmann P , Gorunescu F *et al.* Neural network analysis of dynamic sequences of EUS elastography used for the differential diagnosis of chronic pancreatitis and pancreatic cancer. Gastrointest Endosc 2008; 68: 1086–94. 18656186 10.1016/j.gie.2008.04.031

[38] Saftoiu A , Vilmann P , Gorunescu F *et al.* Efficacy of an artificial neural network‐based approach to endoscopic ultrasound elastography in diagnosis of focal pancreatic masses. Clin Gastroenterol Hepatol 2012; 10: 84–90. 21963957 10.1016/j.cgh.2011.09.014

[39] Marya N , Powers P , Chari S *et al*. Utilization of artificial intelligence for the development of an EUS‐convolutional neural network model trained to enhance the diagnosis of autoimmune pancreatitis. Gut 2021; 70: 1335–44. 33028668 10.1136/gutjnl-2020-322821 PMC13163153

[40] Naito Y , Tsuneki M , Fukushima N *et al*. A deep learning model to detect pancreatic ductal adenocarcinoma on endoscopic ultrasound‐guided fine‐needle biopsy. Sci Rep 2021; 11: 8454. 33875703 10.1038/s41598-021-87748-0 PMC8055968

[41] Ishikawa T , Hayakawa M , Suzuki H *et al*. Development of a novel evaluation method for endoscopic ultrasound‐guided fine‐needle biopsy in pancreatic diseases using artificial intelligence. Diagnostics 2022; 12: 434. 35204524 10.3390/diagnostics12020434 PMC8871496

[42] Kuwahara T , Hara K , Mizuno N *et al.* Usefulness of deep learning analysis for the diagnosis of malignancy in intraductal papillary mucinous neoplasms of the pancreas. Clin Transl Gastroenterol 2019; 10: 1–8. 10.14309/ctg.0000000000000045 PMC6602761 31117111

[43] Kurita Y , Kuwahara T , Hara K *et al*. Diagnostic ability of artificial intelligence using deep learning analysis of cyst fluid in differentiating malignant from benign pancreatic cystic lesions. Sci Rep 2019; 9: 6893. 31053726 10.1038/s41598-019-43314-3 PMC6499768

[44] Machicado J , Chao W , Carlyn D *et al*. High performance in risk stratification of intraductal papillary mucinous neoplasms by confocal laser endomicroscopy image analysis with convolutional neural networks (with video) Gastrointest Endosc 2021; 94: 78–87.e2. 33465354 10.1016/j.gie.2020.12.054

[45] Nguon L , Seo K , Lim J *et al*. Deep learning‐based differentiation between mucinous cystic neoplasm and serous cystic neoplasm in the pancreas using endoscopic ultrasonography. Diagnostics 2021; 11: 1052. 34201066 10.3390/diagnostics11061052 PMC8229855

[46] Jang SI , Kim YJ , Kim EJ *et al*. Diagnostic performance of endoscopic ultrasound‐artificial intelligence using deep learning analysis of gallbladder polypoid lesions. J Gastroenterol Hepatol 2021; 36: 3548–55. 34431545 10.1111/jgh.15673

[47] Kim YH , Kim GH , Kim KB *et al*. Application of a convolutional neural network in the diagnosis of gastric mesenchymal tumors on endoscopic ultrasonography images. J Clin Med 2020; 9: 3162. 33003602 10.3390/jcm9103162 PMC7600226

[48] Minoda Y , Ihara E , Komori K *et al*. Efficacy of endoscopic ultrasound with artificial intelligence for the diagnosis of gastrointestinal stromal tumors. J Gastroenterol 2020; 55: 1119–26. 32918102 10.1007/s00535-020-01725-4

[49] Oh CK , Kim T , Cho YK *et al*. Convolutional neural network based object detection model to identify gastrointestinal stromal tumors in endoscopic ultrasound images. J Gastroenterol Hepatol 2021; 36: 3387–94. 34369001 10.1111/jgh.15653

[50] Seven G , Silahtaroglu G , Seven OO *et al*. Differentiating gastrointestinal stromal tumors from leiomyomas using a neural network trained on endoscopic ultrasonography images. Dig dis 2021; 40: 427–35. 34619683 10.1159/000520032 PMC9393815

[51] Hirai K , Kuwahara T , Furukawa K *et al*. Artificial intelligence‐based diagnosis of upper gastrointestinal subepithelial lesions on endoscopic ultrasonography images. Gastric Cancer 2022; 25: 382–91. 34783924 10.1007/s10120-021-01261-x

[52] Tanaka H , Kamata K , Ishihara R *et al*. Value of artificial intelligence with novel tumor tracking technology in the diagnosis of gastric submucosal tumors by contrast‐enhanced harmonic endoscopic ultrasonography. J Gastroenterol Hepatol 2022; 37: 841–6. 35043456 10.1111/jgh.15780

[53] Yang X , Wang H , Dong Q *et al*. An artificial intelligence system for distinguishing between gastrointestinal stromal tumors and leiomyomas using endoscopic ultrasonography. Endoscopy 2022; 54: 251–61. 33827140 10.1055/a-1476-8931

[54] Huang L , Xu Y , Chen J . *et al.* An artificial intelligence difficulty scoring system for stone removal during ERCP: A prospective validation. Endoscopy 2022; 55: 4–11. 35554877 10.1055/a-1850-6717

[55] Kim T , Kim J , Choi H *et al*. Artificial intelligence‐assisted analysis of endoscopic retrograde cholangiopancreatography image for identifying ampulla and difficulty of selective cannulation. Sci Rep 2021; 11: 8381. 33863970 10.1038/s41598-021-87737-3 PMC8052314

[56] Sugimoto Y , Kurita Y , Kuwahara T *et al*. Diagnosing malignant distal bile duct obstruction using artificial intelligence based on clinical biomarkers. Sci Rep 2023; 13: 3262. 36828831 10.1038/s41598-023-28058-5 PMC9958195

[57] Mayra N , Powers P , Petersen B *et al.* Identification of patients with malignant biliary strictures using a cholangioscopy‐based deep learning artificial intelligence (with video). Gastrointest Endosc 2023; 97: 268–78.e1. 36007584 10.1016/j.gie.2022.08.021

